# The feasibility and acceptability of a psychosocial intervention to support people with dementia with Lewy bodies and family care partners

**DOI:** 10.1177/14713012211028501

**Published:** 2021-06-25

**Authors:** Alison Killen, Darren Flynn, Nicola O’Brien, John-Paul Taylor

**Affiliations:** Translational and Clinical Research Institute, 5994 Newcastle University, Newcastle upon Tyne, UK; School of Health and Life Sciences, 5462 Teesside University, Middlesbrough, UK; Department of Psychology, 5995 Northumbria University, Newcastle, UK; Translational and Clinical Research Institute, 5994 Newcastle University, Newcastle upon Tyne, UK

**Keywords:** Lewy body dementia, Lewy bodies, dementia with Lewy bodies, carer support, group intervention, peer support, dementia support

## Abstract

**Objectives:**

Psychosocial support for people with dementia with Lewy bodies (DLB) and family care partners is frequently lacking, despite the need expressed by those with lived experience. Our objective was to examine the feasibility and acceptability of an intervention designed to build coping capability.

**Design:**

The design was non-randomised with all participants receiving the intervention.

**Setting:**

The setting was a Memory Assessment and Management Service in the Northeast of England.

**Participants:**

Participants comprised 19 dyads consisting of a person with DLB and a family care partner.

**Intervention:**

The intervention was group-based, with weekly sessions attended for up to four successive weeks. It was informed by Social Cognitive Theory.

**Measurements:**

Data were collected on recruitment, attendance and attrition, self-efficacy, mood, stress and participant experience.

**Results:**

Recruitment was achieved with minimal attrition and three successive groups were delivered. Care partners felt more in control and able to cope in at least 3 of 13 areas with 73% feeling this way in eight or more areas. Three themes were identified from post-intervention interviews: people like us, outcomes from being a group member and intervention design.

**Conclusions:**

A DLB-specific group intervention is acceptable to people with DLB and family care partners, and recruitment is feasible within a specialist service. Participation may enhance understanding of this condition and reduce social isolation. It may improve care partners’ coping capability particularly if targeted towards those with low prior understanding of DLB and more stress. Means of evaluating outcomes for people with DLB need further development.

## Introduction

Neurodegenerative dementia is a widespread condition most commonly caused by Alzheimer’s disease (AD). The second most common cause is Lewy body dementia, which comprises dementia with Lewy bodies (DLB) and Parkinson’s disease dementia. Dementia with Lewy bodies is a complex dementia, meaning that multiple investigations and erroneous initial diagnoses, often of AD or depression are not uncommon (Surendranathan et al., 2020). It affects multiple systems with symptoms such as spontaneous alterations in concentration and attention, recurrent well-formed visual hallucinations, and rapid eye movement (REM) sleep behaviour disorder. Other difficulties include gait changes, and autonomic dysfunction, which can lead to severe constipation, orthostatic hypotension, and urinary problems (Donaghy & McKeith, 2014). As a consequence, compared with people with AD, people with DLB have a shorter time until first hospital admission and a higher admission rate (Oesterhus et al, 2020). They experience poorer quality of life (Inskip et al., 2016), and an increased mortality risk (Price et al., 2017).

Furthermore, DLB poses substantial demands on family care partners. The hallucinations and delusions, which are typical even in the early stages are challenging, with persistent delusions commonly relating to infidelity or financial abuse (Mueller et al., 2017). As a result, care partners are themselves at increased risk of depression (Leggett et al., 2011). Compared with AD, care partner burden of people with DLB is rated 30% higher on the Relative Stress Scale (RSS) (Svendsboe et al., 2016). This may be a factor in the shorter time between diagnosis and admission to nursing home, with a mean of 663 days for people with DLB, compared with 1336 days for people with AD (Rongve et al., 2014).

The provision of post-diagnostic dementia care and support is a key priority in many countries and endorsed in the UK by the National Institute for Health and Care Excellence (NICE, 2020a, 2020b) and Scottish Government (2020). The NICE (England and Wales) Dementia Guidelines (2018) assert that people with dementia and their families should be offered psychoeducation and skills training, including information about their dementia subtype. However, dementia interventions typically concentrate on the most common symptomology (i.e. that associated with AD), and rarely address the symptoms of DLB. Consequently, their benefit for this population is likely to be sub-optimal (McDermott et al., 2018).

Psychosocial management of DLB symptoms is particularly important as some pharmacological solutions can lead to the worsening of other symptoms, for example, treatment options for parkinsonian motor disturbances can exacerbate psychotic symptoms (Taylor et al., 2020). A systematic review (Connors et al., 2018) noted a dearth of psychosocial interventions for people with DLB. Many also lacked a theoretical basis, prohibiting identification of the active ingredients or the opportunity for replication. Care partners can also benefit from psychosocial interventions through improvements in well-being, depression and burden (Chien et al., 2011). Dementia specific programmes for family care partners such as STrAtegies for RelaTives (START) (Livingston et al., 2013), which reduced care partners’ affective symptoms and depression have been adapted to reflect the needs of care partners supporting people with Lewy body dementia (Foley et al., 2020). Other approaches have included identifying caregiving benefits rather than focussing solely on deficit and burden (Cheng et al., 2014).

Consideration of this dyadic context of care-recipient and care partner is crucial in the context of dementia, and meta-analysis of psychosocial provision has identified the value of jointly attended interventions (Brodaty and Arasaratnam, 2012). This indicates the potential for utilising this approach in DLB, where family care partners commonly feel under-supported (Killen et al., 2016), and 86% (*n* = 611) feel somewhat or very isolated (Leggett et al., 2011). Given the poor public awareness of DLB (Capouch et al., 2018), opportunities to interact with others in comparable circumstances could be particularly helpful. Social Cognitive Theory (Bandura, 1986) a widely applied evidence-based theory of behaviour change stresses collective agency, making it well suited to group interventions. Self-management approaches based on Social Cognitive Theory can help people understand their illness and build empowerment and coping strategies (Bandura, 1997).

The rationale for the present study was the poor prognosis of DLB (Mueller et al., 2017), and the lack of evidence-based DLB-specific interventions deliverable close to the time of diagnosis, a key stress point with a pressing need for support and information (Newbronner et al., 2013). This ‘needs gap’ was confirmed through insights gathered during consultations and engagement activities with people attending a Lewy body disease clinic, and as part of the Diamond Lewy study on the assessment and management of DLB (Thomas et al., 2018). Key support and information needs were identified from 125 responses to a national online survey hosted by the Lewy Body Society (Killen et al., 2016). Findings concurred with international studies (Zweig and Galvin, 2014); suggesting themes shared a global perspective.

## Method

The study was approved by the Newcastle and North Tyneside 1 Research Ethics Committee, (reference 15/NE/0391). *The aim was to evaluate a co-designed, psychosocial intervention for people with DLB and family care partners. The primary objective was to assess the feasibility and acceptability of recruitment and completion.* Three secondary objectives were to:

1. Identify the most appropriate primary outcome measure to inform a future multi-centre evaluation study.
2. Collect preliminary data on the potential effectiveness of the intervention to change the key component of self-efficacy, a construct linked to higher self-esteem, better well-being and adaptation to chronic diseases (Bisschop et al., 2004; Karademas, 2006).
3. Optimise the intervention content.

Participants were recruited from a specialist Lewy body disease clinic, which received referrals from across the Northeast of England. Inclusion criteria comprised a diagnosis of probable DLB made in the preceding 12 months according to the DLB Consortium consensus criteria (McKeith et al., 2017), and of which the person with DLB and their primary care partner were aware. Exclusions were people awaiting dementia related diagnostic test results, or for whom engagement with group activities would be difficult to support, for example, severe hearing impairment, insufficiently fluent English or challenging behaviour.

Design and development followed the Medical Research Council guidance for complex interventions (Craig et al., 2008). To produce an optimum package and incorporate a strong element of co-design, significant development work took place. This included home-based interviews (*n* = 4), a focus group with people with DLB and care partners (*n* = 8) and two Patient and Public Involvement and Engagement (PPIE) meetings. Findings, which complemented the aforementioned survey, included stakeholders’ views regarding content, format and practicalities such as accessibility. A survey of clinicians experienced in dementia (*n* = 7) confirmed demand, but identified two possible barriers to scalability, a lack of sufficiently knowledgeable health care professionals and deliverability in the economic climate.

Following these consultations, and with the engagement of four experts by experience in an iterative co-design process, a post-diagnostic intervention was developed theoretically informed by Social Cognitive Theory. The targeted psychological construct was self-efficacy, with the enhancement of other key constructs (self-regulation and vicarious learning) also anticipated through engagement in the intervention.

Given the pilot nature and focus on feasibility, the study was non-randomised with all participants receiving the intervention. The facilitators comprised a trainee health psychologist and research nurse, both experienced in supporting people with DLB and family care partners. The delivery site was a hospital memory clinic within the Memory Assessment and Management Service of a hospital in the Northeast of England, an easily accessible venue familiar to the attendees. Travel expenses were reimbursed.

To enable replication with fidelity, description of the intervention is based on the TIDieR (template for intervention description and replication) checklist (Hoffmann et al., 2014). The recruitment target was 18 dyads allocated by recruitment date to one of three groups for successive delivery. Groups were anticipated to be sufficiently small to encourage participation, yet large enough to maintain the benefits of social interaction, despite occasional absences. Sessions were delivered weekly over consecutive weeks with consent and assessment measures completed on arrival at session one. All participants provided written informed consent. Groups one and two comprised three sessions lasting two and a half hours. Feedback including participant interviews was then used to optimise Group three. Participants remained together throughout session one, but for subsequent sessions, people with DLB and family care partners met separately following the mid-session break. Each session concluded with 30 minutes of socialisation and individual conversations with the facilitators. Refreshments were offered on arrival and available throughout. Participants received a telephone reminder the day before each session and an intervention handbook for on-going support. Two former care partners of people with DLB observed one session and provided feedback.

The intervention content was informed by four over-arching themes:

1. Gaining and providing social support
2. Becoming more informed
3. Knowing where to turn
4. Developing or maintaining a positive outlook

Thirteen core topics identified during the development phase were addressed. These encompassed the predominant cognitive, behavioural and physical changes associated with DLB (Table 1).

**Table 1.** Intervention topics.


Sleep disturbances
Managing uncertainty
Feeling part of my family/community
Medications
Feeling positive
Progression and planning
Physical changes
Research opportunities
Visual hallucinations
Fluctuations
Local support and activities
Changes in thinking and memory
Finding out how others cope

**Figure 1.**
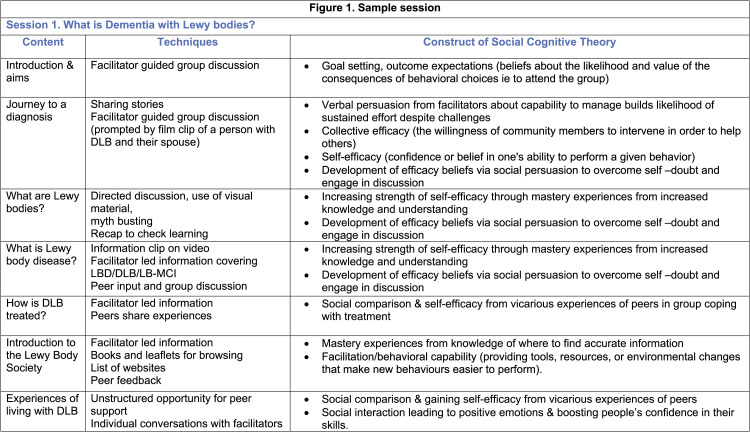
Sample session with key active ingredients.

Positive strategies formed part of each session. These included gratitude diaries, benefit finding and goal setting. Topics covered separately by people with DLB included symptom management, emotional expression and ways of responding to their diagnosis. Care partners addressed disease progression, behavioural challenges and ways to cope and thrive (see Figure 1 for sample session with key active ingredients).

Delivery modes included discussions in groups and within dyads, written expression and drawing. Skills mastery was encouraged through modelling of possible responses by facilitators and peers, with opportunities for practice. Video clips showing people with DLB and family care partners discussing successful strategies for self-management were used to prompt discussion and build self-efficacy through vicarious (observational) learning. Participants were signposted to local voluntary groups, faith groups and charities as sources of support. On-going engagement with group peers was encouraged both directly and through online or phone contact.

Assessment and evaluation were conducted using mixed methods. To meet the primary objective to assess feasibility and acceptability, recruitment and completion rates and any reasons given for attrition were recorded. Each dyad also discussed their experiences in a semi-structured, home-based interview with an intervention facilitator (AK). Interviews lasted 30–45 min and were held 4–12 weeks post-intervention. They were digitally recorded, transcribed verbatim, and uploaded into NVivo12. Thematic analysis was based on the six-phase approach of Braun and Clarke (2006). The data were first analysed independently by a researcher (AK) and two PPIE group members (briefed and working as a pair) to search for potential themes. Transcripts were read and re-read, with similar aspects grouped under nominal headings. Initial codes were then generated, collated into initial themes and examined further. No a priori coding frame was used, although the study aims were kept in mind. Themes were compared and any disagreements discussed and resolved to create the structure of key themes and sub-themes.

To meet the secondary outcomes, participants completed pre- and post-intervention measures of self-efficacy. People with DLB completed the Generalized Self-Efficacy Scale (Schwarzer and Jerusalem, 1995). Care partners completed two measures designed to assess self-efficacy in the context of caregiving, the Family Care Partner Self-Efficacy for Managing Dementia Scale (Fortinsky et al., 2002) and the Revised Scale for Caregiving Self-Efficacy (Steffen et al., 2002). To identify the most appropriate primary outcome measure for future use, questionnaires were examined for missing data and any misunderstandings with the questions, and interview feedback was gathered regarding their acceptability. Participants completed a baseline depression measure (Geriatric Depression Scale GDS-15, Sheikh and Yesavage, 1986). Care partners also completed a 4-point Likert scale evaluation in which they indicated perceived changes in their capability to cope in each topic areas, and pre- and post-intervention measures of the Perceived Stress Scale (PSS) (Cohen and Janicki-Deverts, 2012), and RSS (Greene et al., 1982). Although this was not a powered analysis, post hoc exploratory t-tests were conducted.

Optimisation to inform Group three was undertaken using interview feedback, facilitator and co-facilitator discussions and responses from the session observers. The change process gave precedent to participants’ views, and required amendments to be supported by the majority of participants who expressed a view.

## Results

### Primary outcome

**Figure 2.**
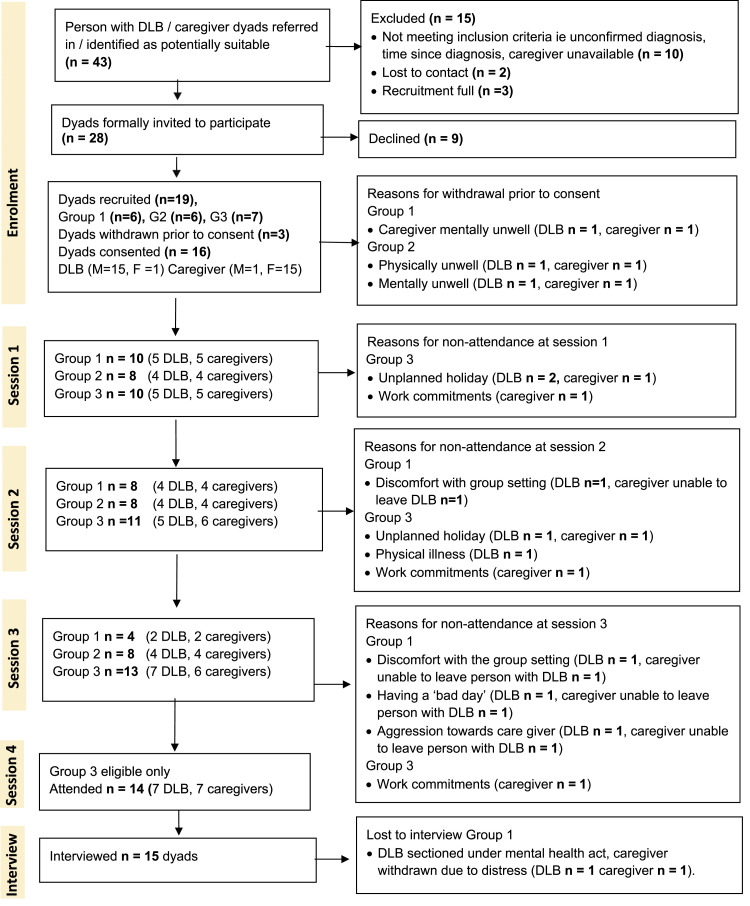
Recruitment flow diagram.

*For the primary outcome, the feasibility and acceptability of recruitment and completion***,** 43 dyads were identified. Of these, fifteen (35%) failed to meet the inclusion criteria, and the remaining twenty-eight (65%) were approached. Nineteen dyads (44%) agreed to participate and were allocated across three groups (see Figure 2 recruitment flow diagram). The intervention was delivered as planned. Groups one and two each comprised six dyads, and Group three comprised seven dyads. Most participants with DLB were male (M =18, F = 1). Participants with DLB had a mean age of 76 years (range 67–88); care partners’ ages were not recorded. The care partners’ relationship to the person with DLB was spouse (*n* = 13), daughter (*n* = 2) and brother (*n* = 1). Three dyads withdrew prior to consent due to illness. The overall attendance of consented participants was 85%, with attrition predominantly attributable to illness or apathy in the person with DLB.

Mild (*n* = 7) or moderate (*n* = 1) depression (GDS-15) was indicated in 50% of participants with DLB, and mild depression was indicated in 25% (*n* = 4) of care partners. Care partners’ scores on the PSS ranged from 0–29 (max 40), with 62.5% (*n* = 10) indicative of moderate or high levels of stress. Care partners’ scores for the RSS, ranged from 4 to 40 (max 60) with 31% (*n* = 5) indicative of a high risk of psychiatric morbidity.

Fifteen dyads were interviewed about their experiences. All care partners and all but one person with DLB described their participation as positive and said they would recommend the intervention to others in their situation. Three themes were identified; people like us, outcomes from being a group member and intervention design (see Supplementary Material for expansion of themes with direct quotations to highlight the participants’ voice).

## People like us

Meeting others in a similar situation was frequently mentioned as highly positive. Care partners described benefiting from solidarity with others in a similar position, peer expertise and the opportunity to compare coping strategies. Several participants with DLB recalled discussions with peers around symptoms, medications and investigations. Social comparison was common and often supportive although occasionally generated negative feelings notably fear of the future.

## Outcomes from being a group member

Participants identified feeling differently about their situation following the intervention. Changes were categorised into sub-themes of ‘feeling more confident to cope’ and ‘feeling more informed’. Feeling more confident to cope was predominantly referenced by care partners, but also mentioned by one person with DLB. This related to managing new symptoms, finding help and using coping strategies. The majority of participants described feeling more informed. For care partners this constituted a greater understanding of the scope of the disease and strategies for managing symptom. Some participants with DLB noted understanding what DLB was for the first time, while others understood how DLB differed from AD.

## Intervention design

Sub-themes related to the intervention design comprised ‘format’, ‘resources’ and ‘barriers’. The format was highly acceptable to the majority of participants who particularly endorsed the session structure, which facilitated meeting their peers in separate groups. Intervention resources were well received, particularly the handbook, gratitude diaries and introduction to mindfulness. Barriers to participation were described infrequently and predominantly in people with DLB. They included apprehension, fatigue, dislike of group settings and physical discomfort.

### Secondary outcome 1: To identify the most appropriate primary outcome measure to inform a future multi-centre evaluation study

The assessment measures were reviewed to determine their suitability including their acceptability to participants. No care partners expressed a dislike of the Family Care Partner Self-Efficacy for Managing Dementia Scale which measures certainty for being able to cope with 10 caregiving situations. Thirteen participants completed both pre- and post-intervention scales with no specific questions unanswered. Three post-intervention scales were uncompleted when the needs of the person with DLB led to the cancellation or curtailment of the appointment. Care partners found the Revised Scale for Caregiving Self-Efficacy difficult to use. Most struggled to express their confidence with the statements in the required percentage format with just four measures fully completed. Several care partners expressed discomfort at completing measures containing care partner burden questions whilst in close proximity to their care-recipient.

No participants with DLB expressed any dislike of the self-efficacy measure generalized self efficacy scale. The completion time was not excessive at around 10 minutes and the facilitators noted no concerns. However, many responses were highly variable, and frequently referenced activities that lacked confirmatory responses from care partners, indicating a lack of insight from people with DLB into their self-efficacy. Even with facilitator support, most were unable to identify changes over the course of the intervention.

### Secondary outcome 2: To collect preliminary data on the potential effectiveness of the intervention to change self-efficacy

**Figure 3.**
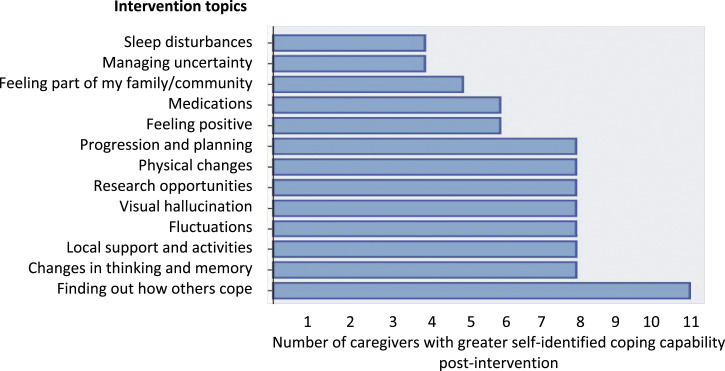
Improvements in coping capability according to topic area.

Care partner scores for the Family Care Partner Self-Efficacy for Managing Dementia Scale were 14–86% pre-intervention and 38–100% post-intervention. There was no significant difference between scores pre-intervention (mean ± SD: 62.00 ± 20.12) and post-intervention (62.69 ± 15.80; t(12) = −0.15, *p* = 0.88). However, care partners with lower pre-intervention self-efficacy demonstrated a significantly greater improvement compared to those who scored more highly (r = −0.64, *p* = 0.02). This included an improvement from 14% to 44% in the lowest scoring participant. Two participants who scored over 80% pre-intervention showed slightly reduced post-intervention scores. On exploratory analysis, there was a significant relationship between change in self-efficacy and PSS scores (r = 0.66, *p* = 0.01), with higher pre-intervention stress associated with a better response in self-efficacy. A similar relationship was noticed with the RSS, albeit not significant (r = 0.38, *p* = 0.21). Care partners also completed a Likert scale response to the question ‘After attending the group, how much do you feel in control of the following situations and able to understand and cope with them, or find help to manage them?’ All respondents (*n* = 11) stated feeling slightly or much more in control and able to cope in at least three of the 13 topic areas, with 73% stating this in eight or more areas. No care partners felt less able to cope in any topic area (see Figure 3).

### Secondary outcome 3: To optimise the intervention content

In accordance with the rationale for change, the following amendments were incorporated into Group three:

1. A reduction in session duration from 2.5 to 2 h, and an increase from three to four sessions

The original three-session structure reflected stakeholder feedback at the planning stage regarding ease of attendance given the multiple challenges associated with DLB, and sustainability within NHS funding constraints. Interview feedback revealed some people with DLB had difficulty sustaining concentration, which necessitated shorter sessions. Increasing the number of sessions allowed delivery of the full content, and provided an additional opportunity for peer interaction.

2. A timetabled opportunity for participants to share their stories

Many people had experienced difficult and prolonged journeys through the diagnostic pathway. The facilitators observed these stories being shared as people introduced themselves. Embedding this into the content increased participants’ familiarity with one another. It formed a cathartic beginning and enabled greater engagement with subsequent elements of the intervention.

3. A shorter interval between attending and evaluation

Conducting the evaluation interview after 12 weeks caused people with DLB some difficulty in remembering details with clarity, reducing this to four weeks enabled greater recall.

4. Increased recruitment by one dyad

Following the withdrawal of three dyads prior to consent in earlier groups, recruiting an additional dyad aimed to maintain sufficient group size for participants to benefit from peer support.

Following Group three, two further amendments were identified for incorporation into future deliveries: firstly, home completion of the care partners’ outcome measures to resolve their concerns about completing them seated beside their care-recipient; secondly, obtaining a recent cognitive score from referring clinicians (i.e. MMSE) to increase group cohesiveness by grouping participants with similar levels of impairment.

## Discussion

This study describes the first multi-component intervention designed to meet the specific psychosocial needs of people with DLB and family care partners*. The primary outcome was the feasibility and acceptability of the intervention for people with DLB and family care partners. The feasibility of delivery within a specialist Lewy body disease service was confirmed through recruitment of the planned sample, and delivery of the intervention to 16 person with DLB and family care partner dyads with minimal attrition of consented participants. Proof of concept was confirmed through positive evaluations. These included rare insights into the views of people with DLB alongside the essential but more typically obtained, care partner perspective.*

*Acceptability was demonstrated by both people with DLB and care partners who overwhelmingly stated having enjoyed attending and chose to attend subsequent sessions*.

The high level of acceptability may reflect the DLB-focussed content, which included disease specific information and strategies to address typical symptoms such as visual hallucinations, sleep disorders and commonly experienced neuropsychiatric features. For example, significantly more depressive symptoms typically occur in people with DLB (73%) compared with AD (56%) (Fritze et al., 2011), such that depression is a supportive clinical feature (McKeith et al. 2017). To reflect this, participants were introduced to strategies engendering a positive outlook such as gratitude diaries (Killen and Macaskill, 2015). Apathy is commonly experienced in DLB, and may impair engagement with treatment (Mueller et al., 2018). In response, participants explored mentally stimulating activities, and opportunities to be physically active within their physical limitations, were signposted to local opportunities for social interaction tailored to their interests, and supported to set attainable activity goals.

The intervention was anticipated to improve self-efficacy through being theoretically informed by Social Cognitive Theory. Whilst all care partners identified an increased ability to cope in a number of areas, the post hoc analysis generated a hypothesis that care partners with lower pre-intervention self-efficacy and higher perceived stress demonstrate a significantly better increase in self-efficacy compared to those with higher self-efficacy and lower perceived stress. This differential effect is worthy of future testing in a larger, powered study. It may indicate that the intervention improves coping capability particularly if targeted towards care partners who have the most to gain, those with low prior understanding of the condition and potentially more stress. Conversely, the reduction in self-efficacy scores noted in care partners with particularly high pre-intervention scores may reflect new concerns about the future, generated through meeting peers coping with difficulties not previously encountered. Although these care partners did also identify their increased ability to cope in a number of areas, these findings highlight the challenging task of preparing care partners and people with DLB to plan for future needs without overwhelming them. Improved access to health practitioners with a comprehensive understanding of DLB is key in building confidence that support and information will be readily accessible as future challenges occur.

Whilst many care partners, particularly those lower in pre-intervention self-efficacy showed increased self-efficacy, following their participation, a reduction in RSS scores was less evident. However, interview analysis demonstrated that many care partners’ experienced changes in their attitudes and feelings, and their understanding about DLB (see Supplementary Material). This provides further evidence of their increased coping capability. It suggests that despite recognising their continuing high levels of caregiving stress, perhaps due to the uncertain day to day variability and fast progressing nature of this condition, care partners felt better equipped to manage. It also highlights the importance of evaluating multi-component interventions using a range of approaches, and further qualitative research in this area is recommended.

While multi-component support interventions improve care partner well-being in the wider population with dementia, the factors mediating this are unclear, particularly as outcomes are evaluated more positively when analysed qualitatively than on using quantitative measures (Dam et al., 2016). The current study supports these findings. A range of self-efficacy scores were evidenced, despite the high level of positive qualitative feedback, alongside all participants referencing being more able to cope in at least three topic areas thus by extension indicating increased self-efficacy. This may reflect the enhancement of a number of constructs, perhaps suggesting the need to measure additional constructs, for example resilience or social support. Although social support is a multi-dimensional concept consisting of structural, functional and evaluative aspects with little consensus which social support measures are most applicable for older adults (Dam et al., 2016), the intervention delivered social support both through participation and as a potential future resource through the exchange of contact details with peers, and information regarding community provision. Providing a safe and supportive environment with reinforcement of care partners existing capabilities, and opportunities to learn vicariously (observation/interaction) from similar others, may also have developed positive outcome expectations when dealing with current or anticipating future challenges. This may have had a role in alleviating caregiving stress, an issue widely acknowledged, and confirmed by the current study where 63% of care partners reported moderate or high stress.

While the support care partners derived from their peers supports existing evidence (Keyes et al., 2016; Smith et al., 2018), people with DLB also greatly valued their first opportunity to meet similarly placed others. This supports findings that established support groups are often not particularly relevant to people with rarer dementias owing to significant differences in age, life situations and symptoms (Brotherhood et al., 2019). In addition, care partners can misconstrue the values and preferences of people with dementia in relation to autonomy and underestimate their decision-making ability (Reamy et al., 2011), despite many developing their own coping strategies based on behavioural and cognitive techniques, which they can find it empowering to share (Collerton and Dudley, 2004; Diederich et al., 2003). The modelling of peers in the current study successfully addressing common DLB challenges including excessive daytime sleepiness and identifying misperceptions may have increased others’ self-belief in their own capability. As people with DLB generally retain insight into their condition for longer compared with people with AD (Zweig and Galvin, 2014), this may have increased their likelihood of engagement with, and benefit from the group activities. Further benefit may have resulted from promoting a strong social element of universality of experience with problems being similar to others rather than unique (Yalom and Leszcz, 2005).

The positive perspective of activities such as goal setting encouraged participants with DLB to maintain activity and identify personal strengths. This supports previous findings identifying the benefits of embracing strengths and focussing on activity (Mountain, 2006). The opportunity to plan pleasant events and share successes may have provided a welcome change in emphasis for people with DLB compared to the passivity of receiving assistance for activities once easily conducted and the potentially distressing recounting of their difficulties to health and social care professionals.

The separately facilitated provision, which enabled conversations that could be difficult with the other member of the dyad present, did not solely benefit care partners. Assumptions may be made regarding how people with DLB are impacted for example suggesting particular symptoms such as visual hallucinations are distressing, when these may actually be more problematic to care partners (Barnes et al., 2013). Time apart provided a novel opportunity for people with DLB to identify their own key concerns. These included needing additional time to respond to questions, difficulties participating in family conversations, and uncertainty over the veracity of unpleasant vivid dreams. Through an emotional expression activity, they shared feelings of embarrassment at asking for help, anger over having their driving licenses removed, frustrations when family members completed their sentences and distress at feeling a burden. People with dementia may perceive themselves (or be perceived) as no longer able to contribute to society in a meaningful way (Moyle et al., 2010). Exploring solutions together and having concerns and feelings validated by their peers could help address the previously discussed poorer quality of life in this population (Figari-Jordan et al., 2012).

The limitations to generalisability inherent in conducting this development study in a region where DLB is well recognised and specialist services are in place are acknowledged. However, although specialist DLB services are sparse, only being accessible to an estimated 5% of people with DLB within United Kingdom (UK) memory services, this may expand with the cohort anticipated to increase following the recently developed DLB assessment and management toolkits (Thomas et al., 2018; Taylor et al., 2020). We would argue that a bespoke intervention is therefore possible within routine services. A typical memory clinic seeing 500 patients a year might expect to diagnose 20–30 people with DLB, which would enable delivery of four to five groups per year. Such numbers are sufficient to indicate a need, but not too overwhelming to be embedded into the programmes of psychosocial activities typically run in memory clinics, and which predominantly cater for people with AD.

People with DLB were unable to complete the self-efficacy measure selected for the current study and new measures of this construct may need developing for people with dementia (Moniz-Cook et al., 2008). However, this may not be the most appropriate evaluative tool. Øksnebjerg et al., (2018) suggest that greater use of ‘in the moment’ evaluations better reflect the beliefs of people with dementia that recording outcomes and reactions during and at the end of activities are the most authentic, most accurately reflect what they think and feel, and are easiest to manage. In the current study, people with DLB responded verbally and through affirmative gestures. Body language and reactions could be used as measures of engagement by session observers with checklists. Methodology could also include audio recording.

This study demonstrates that a theory and evidence-based psychosocial intervention uniquely focussed on DLB can be recruited to and delivered within specialist services, and is acceptable to people with DLB and family care partners. It has generated hypotheses about the potential effectiveness and mode of action of the intervention that warrant further attention. Directions for future research include the design of an intervention workshop to support delivery by health care professionals in generalist memory services. This would enable a large scale study of effectiveness to be conducted with control group comparison, and the identification of additional constructs contributing to the positive evaluations. Provision of this brief, intervention in the early stages of this complex disease could mitigate some of the difficulties, which generally increase as the condition progresses, facilitating adjustment to the diagnosis, and enabling people to seek out appropriate services and benefit from effective coping strategies, including peer support.

## Supplemental Material

sj-pdf-1-dem-10.1177_14713012211028501 – Supplemental Material for The feasibility and acceptability of a psychosocial intervention to support people with dementia with Lewy bodies and family care partners Click here for additional data file. Supplemental Material, sj-pdf-1-dem-10.1177_14713012211028501 for The feasibility and acceptability of a psychosocial intervention to support people with dementia with Lewy bodies and family care partners by Alison Killen, Darren Flynn, Nicola O’Brien and John-Paul Taylor in Dementia
