# Supplementary material for: The feasibility and acceptability of a psychosocial intervention to support people with dementia with Lewy bodies and family care partners
Source: Dementia (London). 2021 Jun 25;21(1):77–93. doi: 10.1177/14713012211028501 (PMC8721619; doi:10.1177/14713012211028501)
Supplement: sj-pdf-1-dem-10.1177_14713012211028501 – Supplemental Material for The feasibility and acceptability of a psychosocial intervention to support people with dementia with Lewy bodies and family care partners [file sj-pdf-1-dem-10.1177_14713012211028501.pdf]

Themes from participant interviews

Supplemental material for "The feasibility and acceptability of a psychosocial intervention to support people with Dementia with Lewy bodies and family care partners".

Theme	Participants' responses
**1. People like us**	Participants were unaware of anyone else with DLB within their community or social network. Several people with DLB observed that this made them feel lonely and it was a relief to find that there were others who shared similar experiences.<br><br>*It is valuable to meet with people of a similar condition. I mean, that was the striking thing; the single striking thing was seeing people in a similar position, saying what their problems and thoughts were.* (Paul, 81 with DLB).<br><br>Caregivers stated meeting others was positive because they benefited from their peers' expertise including lifestyle adaptations, and the chance to compare coping strategies for managing challenges such as hallucinations. They exchanged information regarding local services including subsidised transport, 'games for the brain' and seated exercise classes. They forged links for future mutual support through exchanging contact details.<br><br>*I found it was interesting, just sharing other people's symptoms, because they do vary quite a lot, but some are, obviously, the same. It was good to hear that other people were having, or have had, the same symptoms. I think it's good to know that there are other people out there. You feel as though you're not on your own, even though it could be awful and it could be awful for them. But it helps to know that it's not just you.* (Margaret caregiver)<br><br>As a result of meeting people who had or were caring for someone who had DLB for the first time, social comparison was common both between caregivers observing what situations their peers were facing and between the participants with DLB. One participant with DLB described finding it comforting that others seemed more adversely affected than he was. However, negative feelings were described at times. One person with DLB found listening to other peoples' 'sad stories' was difficult while another noted:<br><br>*Because I was diagnosed early, I suppose, they seemed to be in a slightly more advanced state of Lewy bodies… It made me depressed a bit, to think I'm going to be like them in a few years' time. I could just foresee what sort of problems other people have, and that was a bit of a downer.* (Doug, 72 with DLB)
**2. Outcomes from being a group member**<br><br>**i. Feeling more confident to cope**	Caregivers frequently referenced feeling more able to cope following participation in the intervention. This included feeling more capable of recognising new symptoms and understanding that these were part of the condition and possible treatment and support options.<br><br>*To see the variety of symptoms was quite interesting and, to an extent, I mean, obviously helpful, slightly unnerving because some of them were frightening. I think the ones where the people were walking along, seeing a car coming towards them and suddenly it was two cars and one was going into the other, I mean that's a frightening manifestation of the disease. But, if something like that happened, I wouldn't panic, now, I would be able to say, "This is part of the disease, we just have to see if there's anything that can*

	make it better," like being less tired or whatever. (Christine, caregiver).<br><br>Talking to peers who had accessed support from a variety of community and statutory sources increased caregivers' confidence through knowing where to find help to cope with their caregiving responsibilities. Other mentioned having gained confidence to ask for help<br><br>*I go out now and I find what I need; I know there's going to be somebody somewhere to help me, where I didn't know that before I came to the group, that steered me to go looking for help, which we have done.* (Grace, caregiver).<br><br>One caregiver identified how talking to peers during the group was the first time she had discussed her feelings and caregiving situation but having started this had enabled her to continue doing this. She described that opening up more and talking rather than keeping things 'bottled in' made it easier for her to cope. Several caregivers reported using benefit-finding techniques, prompted by the gratitude diaries started in-session to help them cope through identifying positive aspects within their lives.<br><br>*The good thing diary is good; I don't fill in the diary, but when I go to bed, I think about the good things. Sometimes there's none, but sometimes there's more than three,* (Maude, caregiver)<br><br>One participant with DLB said that he had found accepting his diagnosis very difficult but that attending the group had helped him feel more able to cope with the symptoms he was experiencing. He attributed this to a better understanding of what DLB was and what to expect.<br><br>*Well, yes, it helps you to cope with it, but I think it's just having more understanding that that's not abnormal, it's part and parcel of the symptoms.* (Isaac, 73 with DLB).
**ii. Feeling more informed**	Several participants with DLB expressed how previously their understanding of the condition had been minimal. They described a greater understanding of what DLB comprised, having previously often received more information about what it was not (eg not Alzheimer's, not Parkinson's). A few people observed that the explanations they received could be repeated to friends and family who asked about the condition. One described not being so much in the dark, while another noted<br><br>*Lewy body? Really, you just didn't know what it was but now you know exactly what it is* (Ted, 74 with DLB).<br><br>Opportunities for people with DLB to become more informed included conversing with other caregivers and with peers. Information emanating from people with lived experience of particular situations was deemed highly credible<br><br>*I've just been recommended a change in the drugs I was going to get, and the lad, I was sitting next to, well, he was sitting with his wife, she said "Well, D. is on that now," and I carried on talking to D. about it, because it's somebody who was using it. It just got me thinking… "Did he have any trouble with them? Do they keep you awake at night?" You know simple things. You*

	know, it's not as if it's coming from a doctor all the time. It's Joe Bloggs who's come onto the same course as you. (Tom 74 with DLB).<br><br>Caregivers described the importance of the intervention for adding to their knowledge of DLB. One observed that it was much more than simply getting to know a few people in similar situations but that they had received very good information including where to get help and at what point. Another caregiver observed<br><br>*I was very impressed, and I thought the information that was given was superb. I think it answered a lot of questions, it put a lot of symptoms into the right place as well.* (Kath caregiver).<br><br>Several also noted having a greater understanding of why their care-recipient behaved as they did and how different ways of responding could have beneficial or negative outcomes. Few had previously had any understanding of the range of autonomic and mobility difficulties which form part of DLB. They found it enlightening to link previous disparate symptoms and to understand more about when to report changes and seek help.<br><br>*I do understand a bit more about how it works, and although D's symptoms are not extreme, he has changed in different ways, and I realise that it's part of his illness, and he can't help the things that he does* (Gloria, caregiver)<br><br>Caregivers described their increased understanding of DLB, particularly the range of domains impacted, and awareness of when signs of infection or medication side effects were significant and warranted intervention. Others benefitted from understanding that behaviour changes such as apathy, false beliefs and cognitive fluctuations were attributable to DLB rather than a true representation. A number noted that their improved understanding would enable them to explain the condition to friends and family. Several commented that the intervention filled an information and support gap, as while dementia and carer related charities they had contacted were aware of DLB, staff had little specialised knowledge.<br><br>*There is very little about any specific condition. So far, you know, when I've been to, like, to Carers N., or speaking to the Alzheimer's, they don't know enough about the Lewy bodies. They know that there are different conditions, but there's no specific one that they can, you know, talk to you about.* (Agnes, caregiver).
**3.**<br><br>**Intervention design**<br><br>**i. Format**	The group based face-to-face format of the intervention was popular with both people with DLB. It provided a social element and removed pressure to respond which may have occurred with a one to one intervention.<br><br>*You can weigh more situations up at once in a short time with a group. Whereas when you're one-to-one like this, you can only go at a certain pace, can't you? While somebody else is talking, you can see what other people's reactions are to them in the group, which you can't do in a one-to-one. Yes, I'd recommend that people go to the groups, even if they have fairly mild symptoms like mine, at fairly initial stages. I would say yes.* (Doug, 72 with DLB).

	One caregiver explained that the participants had gelled well together in the group, and that lots of information had emerged from other people's experiences that they would not have gained from the handbook alone. In addition to describing the group as 'interesting and informative', she stated how enjoyable she had found talking to other participants.<br><br>*I think the group is better because everybody has different things to add to the pot so that we get more and more information out of it and you get more and more things. With the group you can pass information to each other and learn from different ones, what they are telling you and things like that, finding different places out and different things*. (Lily caregiver).<br><br>Only one person with DLB and no caregivers would have been as happy to receive the content on an individual basis. Several participants stated the importance of the face-to-face aspect and that they would have been unlikely to access similar content had delivery been online.<br><br>The format comprising people with DLB and caregivers meeting separately for part of each session apart from session 1 was overwhelmingly endorsed. One caregiver described how she did not want to think about the future while her husband with DLB wanted as many details as possible about what might happen. This was difficult as they attended hospital reviews together. She found it helpful that he could receive answers to his questions during the split part of the session when she was elsewhere. Another participant with DLB explained that this time had it allowed him *'to go and investigate it a bit more myself.'* Several caregivers (interviewed separately from their care-recipient) said they had used the opportunity to express their burden of caregiving within an empathetic environment. This was a sentiment that they would never have expressed within the hearing of their partner with DLB or other family members.<br><br>The engagement and enjoyment of people with DLB while meeting separately from their caregivers was evidenced by several participants. Despite the intervening weeks between the intervention and interview, anecdotes shared during this time were recounted.<br><br>*Time used to fly. It was very good. We had quite a few laughs. That guy who pushed the bloke in the Tyne* (Rob, 74 with DLB).<br><br>Many caregivers noted that their care-recipient had benefited from this separation. One expressed surprise on hearing how engaged their care-recipient had been without her supportive presence.<br><br>*Every time I came back, he was smiling and laughing and I thought, "Well, he's enjoyed that," just by his face and I thought, "Oh, he's been talking."* (Grace, caregiver).<br><br>The variety of delivery techniques was commended. Video clips of a caregiver and their spouse with DLB discussing their experiences were particularly memorable. A number of participants commented favourably on the positive strategies incorporated into each session. They found the encouragement to explore alternative ways of looking at their

	situation was helpful.<br><br>*I got a lot more out of it than I thought I would. I didn't quite know what to expect; I've never been on a thing like that before. It was much more positive than I thought it was going to be* (Doug, 72 with DLB).<br><br>Participants universally supported the mid-morning start time, which allowed for commonly experienced delays and difficulties in starting the day. The group size was widely endorsed as small enough to get to know people and providing opportunities to be heard. One caregiver noted that people with DLB may find a larger group confusing and be more reluctant to speak. Various preferences were expressed regarding the optimum number of sessions with three as a minimum. A number of participants favoured adding more sessions, or occasional updates.
**ii. Resources**	The intervention handbook was identified as a very useful resource. Caregivers valued it as a source of new information as well as for some participants' aspects to reinforce information accessed online. Information provided identifying local sources of community support were valued. One caregiver described using the handbook to access further information about strategies used during the intervention.<br><br>*We read it the first few weeks. That's how we remembered the thing about the thinking positive things before you go to bed* (Gloria, caregiver).<br><br>People with DLB found the handbook particularly beneficial with several noting that they preferred printed information and lacked confidence or trust in online alternatives.<br><br>*It wasn't an 'Idiot's Guide To', but it was ideal for understanding. I wouldn't go on the internet because every Tom, Dick, and Harry can be writing some wonder things which are not true* (James, 73 with DLB).<br><br>For one participant with DLB, reading the handbook gave him plenty of time to take information in whereas when listening, this was soon forgotten. Seeing his symptoms described in print helped him to acknowledge his diagnosis, which he had previously questioned. Another noted that sometimes further questions came to mind long after a conversation and he could consult the handbook for clarification in his own time.
**iii. Barriers**	Several people with DLB described being apprehensive before the first session and having required encouragement. This related to unfamiliarity with group situations, wariness about what they might be expected to do, and reluctance about trying new things. One commented that he needed *'that little bit of a push to get me to go'*.<br><br>One person with DLB expressed discomfort with the group setting and did not attend following the first session. Other barriers to engagement identified by people with DLB included anxiety regarding their perceived poor conversational skills, self-consciousness over their slow responses, and physical discomfort.<br><br>*It's a long time to sit. I thought it was a bit long. I ended up with pins and*

	needles. I want a cushion on my back, I tell you (Bob, 75 with DLB)<br><br>One caregiver found the first session which was preceded by completion of the outcome measures was tiring and less beneficial than subsequent sessions<br><br>*I think, looking back on it, the first one, there was so much packed into it because of the questionnaires that, in fact, I couldn't take as much from it. I found the latter sessions better because of the splitting up and having the two separate groups, where the women were together. I know that those who are in support wouldn't always be women, but we just happened to be, and I found those very useful. And just moving for that, changing after an hour and having a different focus, that's very helpful, so those were less tiring* (Christine, caregiver).<br><br>Participants did not refer to keeping in contact following the group despite most having exchanged details. One couple described having enjoyed meeting again at a clinic appointment and a caregiver noted feeling reassured that she had contact details available for if she needed them. Barriers identified included reluctance of the care-recipient, busy lives and geographical distance, a factor mentioned by several caregivers who were no longer driving.<br><br>*As far as keeping in touch with everybody, I think, yes, if you feel inclined to do that. But I think we've all got our own private lives, and things going on in our lives, that it's just going to complicate matters more. And if they lived closer, if we all lived a bit closer, yes maybe that would have been a good idea, meeting up. But we all live such a distance apart, so I don't think that's quite, you know, I don't think that's feasible.* (Agnes, caregiver).
